# Trajectories in life satisfaction before and during COVID-19 with respect to perceived valence and self-efficacy

**DOI:** 10.1007/s12144-022-03829-x

**Published:** 2022-10-12

**Authors:** Jantje H. de Vries, K. T. Horstmann, P. Mussel

**Affiliations:** 1 Division for Personality Psychology and Psychological Assessment, Institute of Psychology, Freie Universität Berlin, Habelschwerdter Allee 45, 14195 Berlin, Germany; 2 Department of Psychology, University of Siegen, Adolf-Reichwein-Straße 2, 57076 Siegen, Germany

**Keywords:** Covid-19, Life satisfaction, Critical life events, Self-efficacy, Set point theory

## Abstract

Actions taken by governments to counteract the spread of the COVID-19 pandemic led to profound restrictions in daily lives, especially for adolescents and young adults, with closed schools and universities, travel restrictions, and reduction in social contacts. The purpose of the current study is to investigate the development of life satisfaction with assessments before and during the pandemic, including separate measurement occasions during a strict lockdown and when the implemented restrictions were relaxed again. Data are based on the German Personality Panel (GePP) with 1,920 young adults, assessed on four measurement occasions over a period of three years. Using latent change score modeling, we investigate the outbreak of the COVID-19 pandemic with respect to its perception as a critical life event over time. Further, we examine the influence of self-efficacy on change in life-satisfaction, as the belief in one’s innate abilities has been shown to promote health related behavior and buffers against effects of negatively perceived critical life events. While average life satisfaction remained stable across time, we found a main effect of perceived positive valence and self-efficacy on latent change in life satisfaction at the within person level. Expressions of self-efficacy did not moderate the influence of the perception of the pandemic on self-reported life satisfaction. This study provides an important contribution to the recent COVID-19 literature as well as to the debate on stability and change of self-reported life satisfaction.

With the outbreak of the new strain of the coronavirus (COVID-19), the severe measures to counter the spread of the virus led to unprecedented changes in the daily life of people all around the world. From spring 2020 onwards, in many countries all over the world general mobility was restricted, schools and universities were closed, social interactions were limited to the bare necessity, and health regulations such as wearing masks as well as social distancing rules were established (e.g., Ren et al., 2021; Rogowska et al., 2020; Tran et al., 2020; Zacher & Rudolph, 2021). Declared as a global pandemic by the World Health Organization (WHO), the coronavirus pandemic constitutes a collectively experienced environmental impact that displays a severe, large-scale impact on the population in various aspects. The coronavirus pandemic can be interpreted as a collective life event: It leads to struggles that affect many if not most people and demands major adjustments and adaptive behavior (Wundrack et al., 2021). Moreover, critical life events are often defined as transitions that mark the beginning or the end of a specific status (Luhmann et al., 2012). Importantly, events can be perceived very differently (Luhmann et al., 2020) and it has been argued that it is the perception of a specific event and its interpretation that makes it a critical life event, as opposed to its "objective nature". The coronavirus pandemic represents such an event which is often experienced very differently. Further, it has been argued that the preventive measures to combat COVID-19 have a huge impact on psychological well-being, such as life satisfaction. Self-reported life satisfaction reflects individual’s evaluation of one’s own life circumstances and displays an overall assessment of feeling and attitudes about one’s life. Life satisfaction has been shown to decline due to the measures to prevent the spread of COVID-19 (e.g., Ammar et al., 2020; Arslan et al., 2021; Gawrych et al., 2021; Kimhi et al., 2020; Meyer, et al., 2021; Zacher & Rudolph, 2021) but longitudinal investigations with assessments before the beginning of the coronavirus pandemic are still rare. Importantly, it could be the case that reported short-term changes in life satisfaction or retrospective reports of life satisfaction before the pandemic are biased (Buecker & Horstmann, 2021). The current study aims at addressing this gap by providing results from a longitudinal study with assessments before and during the pandemic, including separate measurement occasions during a strict lockdown and when the implemented restrictions were eased again. Moreover, we focus on variables that are theoretically relevant for inter-individual differences in trajectories in life satisfaction. Here, we especially highlight the potentially moderating role of self-efficacy. The personality trait self-efficacy has been increasingly under investigation with regard to the processing of critical life events and might influence how individuals deal with the implementations of the pandemic.

## The impact of anti-COVID-19 measures on adolescents and young adults

With respect to their social consequences, the measures to prevent the spread of COVID-19 pose a strong challenge for adolescents and young adults. Adolescence and young adulthood are demographically and subjectively distinct life stages that reach from the teens through the twenties and are characterized by identity forming and mastering developmental milestones such as gaining independence from parents (Arnett, 2000; Bleidorn, 2015). Restrictions due to the coronavirus pandemic, for example, school and university closures, meant that adolescents and young adults were forced to learn from home and adapt to online video conferencing immediately and mostly without training. Likewise, parent-adolescent relationships were influenced by the anti-COVID-19 measures (Buelow et al., 2021).

Importantly, the consequences of such measures were stronger for some than for others (Buelow et al., 2021; Flesia et al., 2020). For example, children and adolescents with low socioeconomic status, low parental education and migrant status (e.g., Ravens-Sieberer et al., 2021), or previous symptoms of anxiety or depression (Le et al., 2020), were particularly burdened by the effects of the coronavirus pandemic. Around the world, numerous studies reported a severe, negative impact of the preventive coronavirus measures on mental health of adolescents and young adults (Nicola et al., 2020; Ren et al., 2021, Rogowska et al., 2020; Xiong et al., 2020; Zacher & Rudolph, 2021). Specifically, pre-existing inequity across families, e.g., with respect to resources for virtual learning environments, were shown to moderate the effect of anti-COVID-19 measures on learning outcomes (Buelow et al., 2021; Flynn et al., 2021; Fontenelle-Tereshchuk, 2021; La Rosa & Commodari, 2021). In the long run, this will mean that later higher education and consequently the future of young adults might be affected (Engzell et al., 2021).

Along with stay-at-home orders during the coronavirus pandemic, some young adults may have been increasingly exposed to abuse and neglection. Recent research suggests that due to the circumstances of the pandemic, general vulnerability in children and young adults increased while stressors in (working) parents and caregivers also increased–whereas a reduction of normal protective services was noted (Fosco et al., 2021; Ravens-Sieberer et al., 2021; Tso et al., 2020).

Regarding social contact, young adults depend on their worship community with nonfamily members and peer groups probably more than any other age group (Sander et al., 2017). With cancelled events like graduations and proms, important critical life events may have been missed. Moreover, clubs and bars were closed and meeting with friends was nearly impossible. Accordingly, the feeling of loneliness increased, and the quality of social relationships was perceived worse during the pandemic than before (Buecker & Horstmann, 2021). Together, young adults’ social, emotional, and mental well-being may have been burdened by the coronavirus pandemic in various aspects and as a consequence, implementations in daily life might also have interfered with young adults’ perceived overall life satisfaction.

Importantly, recent research concerning the perception of critical life events such as the coronavirus pandemic, has shown that subjective perceptions of life events can better explain the consequences of the life event compared to an objective assessment of the life event alone. For example, introverted young adults might have encountered the restriction of social contact less decisive than extroverted individuals, who were formerly meeting up with their peers on a daily basis. This differentiation has often been neglected in previous research, for example, when investigating related personality change and entering work life /retirement (Asselmann & Specht, 2021), parenthood and paid employment (Denissen et al., 2019) or life events such as beginning a relationship and studying in university (Leikas & Salmela-Aro, 2015). However, there is ample evidence that life events are perceived quite differently because they interact with, for example, preexisting characteristics and attitudes of a person, and thus evoke differences in related personality change (Bleidorn et al., 2020; de Vries et al., 2021; Haehner et al., 2021; Luhmann et al., 2020; Rakhshani et al., 2021). Therefore, the present study aims at addressing this gap by investigating interindividual trajectories in life satisfaction with respect to the perception of the preventive COVID-19 measures–separately assessed for perceived positive and negative valence.

## Trajectories in life satisfaction

Self-reported life satisfaction reflects a subjective overall assessment of feelings and attitudes about one’s life (Fujita & Diener, 2005). High levels of subjective life satisfaction were shown to facilitate many advantageous aspects of life such as longevity (Danner et al., 2001), marriage (Mastekaasa, 1994) and physical condition (Mroczek & Spiro, 2005). Moreover, subjective life satisfaction is often regarded as an important mental health index (Koivumaa-Honkanen et al., 2004) and, thus, is highly desirable in itself.

Several theories have been developed to explain when and why subjective life satisfaction changes or remains stable. One common approach is to try to explain trajectories in life satisfaction in connection with experiencing critical life events (e.g., Andrew et al., 2008; Fujita & Diener, 2005; Lucas et al., 2004). For example, according to Set Point Theory, subjective life satisfaction has a baseline for each individual and the subjective well-being fluctuates around this stable set point (Headey & Wearing, 1989; Lykken & Tellegen, 1996). After experiencing unusual or critical life events, individuals show altered levels of life satisfaction which quickly return to their set point (see Diener et al., 2006, for a review). For a long time, Set Point Theory was the most widely accepted and empirically validated theory of life satisfaction. However, recent research suggests that critical life events can influence life satisfaction lastingly and perturb individuals away from their stable set point without returning to it. For example, in a study by Headey and Muffels (2018), 14–30% of a large German panel recorded medium- and long-term changes in their set points due to various aspects such onset of a chronic health problem and long-term unemployment. Moreover, critical life events like marriage and disability (Anusic et al., 2014), unemployment (Lucas et al., 2004), or widowhood (Yap et al., 2012) seem to have a rather strong influence on long-term levels of subjective life satisfaction. Despite meaningful life events, van Praag and colleagues (Praag et al., 2003) also identified health, family, and finances as important determinants of overall life satisfaction. Hence, external circumstances, such as living conditions and, e.g., reduction of income due to COVID-19 (Tran et al., 2020), might matter more than previously expected and life satisfaction can and does change for some people permanently (Fujita & Diener, 2005). Conjointly, possible adaptations of Set Point Theory are currently under debate because an adequate theory of subjective life satisfaction should account for all factors–for those that tend to stabilize and those that alter (Headey & Muffels, 2018).

## Changes in life satisfaction due to anti-COVID-19 measures

The coronavirus pandemic represents not only a major medical and economic crisis, but also impacts people all around the world on a psychological dimension because of the far-reaching restrictions on daily living (WHO, 2020). This stands in line with most of the recent studies which report declines in subjective well-being due to the implementation of the anti-COVID-19 measures (Ammar et al., 2020; Arslan et al., 2021; Gawrych et al., 2021; Kimhi et al., 2020; Meyer, et al., 2021; Tran et al., 2020; Zacher & Rudolph, 2021). Foremost, people displayed declines in average life satisfaction and positive affect with the onset of the restrictions, in March 2020 (Arslan et al., 2021; Zacher & Rudolph, 2021). Importantly, some studies isolated specific causes of declines in life satisfaction, namely, reduced social participation (Ammar et al., 2020) due to forced social distancing and home confinement (Gonzalez-Bernal et al., 2021; Wang et al., 2020), the concern about the possible infection or dead of loved ones (Arslan et al., 2021), and exhaustion of employees (Meyer et al., 2021). Similar effects on self-reported life satisfaction have been found across different cultures (Gonzalez-Bernal et al., 2021; Meyer et al., 2021; Raza et al., 2020) which underlies the relevance of the topic.

At the same time, several studies have identified external and internal resources to promote resilience with respect to a decline in life satisfaction. Among external resources such as financial independence or good health (van Praag et al., 2003), there are also internal psychological resources which might act as a buffer against distress and promote well-being. For example, high levels in self-efficacy have been shown to negatively relate with mental health problems such as depression, anxiety, and perceived helplessness (e.g., Bandura, 1977; Benight & Bandura, 2004; Luszczynska et al., 2005). Moreover, internal control beliefs have been shown to function as an important coping resource in the face of processing psycho-social stressors (Benight & Bandura, 2004; Luszczynska et al., 2009). Thus, self-efficacy represents a promising source to further investigate interindividual differences in life satisfaction with respect to the implications of the coronavirus pandemic.

## The moderating role of self-efficacy

Self-efficacy describes the expectation of a person to be able to successfully perform desired actions based on their own competencies and to sustain and regulate cognitive, motivational, and affective processes (Bandura, 1977). The influence of self-efficacy on health-related components as well as subjective general well-being has been previously shown across different samples and cultures (e.g., Bandura, 1977; Benight & Bandura, 2004; Luszczynska et al., 2005).

With respect to the coronavirus pandemic, self-efficacy could play an important role towards the processing of a critical life event and related repercussions for subjective life satisfaction. With the onset of the preventive COVID-19 measures, young adults were forced to adapt to new situations such as homeschooling, acquire knowledge about digital platforms, adjust their way of learning and manage their social life in altered ways. This rearrangement of living circumstances represents a time of great uncertainty which might evoke the feeling of loss of control and distress. Accordingly, individuals with low levels in self-efficacy might experience the implications of the pandemic overwhelming and get the feeling of disappointment when, for example, they fail to adjust to new learning tools or lose their connection to peer communities. This, in turn, might echo on life satisfaction levels, as subjective life satisfaction captures a main dimension of well-being related to psychological factors. Together, self-efficacy could act as a protective factor to the processing of the implications of the pandemic and prevent from a decline in life satisfaction.

Previous studies on internal locus of control, which refers to the belief that the outcome of events in one’s life is contingent upon one’s actions, was found to serve as a protective factor. For example, Krampe and colleagues (Krampe et al., 2021) investigated whether locus of control moderated the relationship between COVID-19 stress and general mental distress. Here, internal locus of control served as a buffer to related COVID-19 stress whereas external locus of control exacerbated this relation (Krampe et al., 2021). Likewise, a recent study by Flesia et al. (2020) identified internal locus of control along with emotional stability as protective factors against the level of perceived stress during the coronavirus pandemic. Vice versa, external locus of control has been shown to predict higher anxiety and depression severity after experiencing negative life events (Hovenkamp-Hermelink et al., 2019).

Taken together, self-efficacy constitutes a relevant personality trait for retaining and promoting mental health which is related to controllability appraisals and active coping that might account for individual differences in life satisfaction. However, evidence on similar constructs is still inconclusive and only few studies have provided data before the pandemic to account for a comprehensive picture on the influence of the anti-COVID-19 measures.

## The present study

The preventive measures to reduce the spread of COVID-19 have a far-reaching impact on the subjective well-being of adolescents and young adults, and the prolonged impact on trajectories in life satisfaction has not yet been examined sufficiently. The purpose of the present study is to investigate the influence of the coronavirus pandemic as a critical life event on subjective life satisfaction in Germany, where the anti–COVID-19 measures were imposed from March 2020 onwards. In this study, we focus on individuals who are particularly affected by the pandemic with restrictions in everyday life such as closed schools and universities, travel restrictions, closed nightlife, and reduction in social interactions. We propose that these restrictions lead to change in self-reported average life satisfaction. Our data give the possibility for a holistic view to investigate change in life satisfaction in a representative sample of young adults on four measurement occasions.

As our first hypothesis we argue that life satisfaction should decline with the onset of the anti-COVID-19 measures. Therefore, we investigate trajectories in life satisfaction with assessments before and during the pandemic, including separate measurement occasions during a strict lockdown and when the implemented restrictions were relaxed again. Second, we employ a dimensional approach to critical life events and propose that the perception of the coronavirus pandemic should predict interindividual changes in subjective life satisfaction. Specifically, we expect that experiencing the coronavirus pandemic as more negatively and less positively is related to stronger decrease in subjective life satisfaction compared to individuals who perceive the pandemic less negatively and more positively. Moreover, a possible decline in life satisfaction should be observed at the onset of the restrictions. In line with Set Point Theory, life satisfaction should then return to the original state when restrictions are lifted again.

As a third hypothesis, we expect that self-efficacy moderates the effect of the coronavirus pandemic and its perception on subjective life satisfaction. Specifically, individuals high in self-efficacy should be less negatively affected by the anti-COVID-19 measures with regard to their self-reported life satisfaction. High levels of self-efficacy are thus a protective factor that could buffer the effect of the coronavirus pandemic on subjective life satisfaction.

## Method

### Data and recruiting procedure

We used data from the GePP (Mussel, 2021), a large-scale longitudinal study, starting in 2016. We established the panel in cooperation with a German company that provides a nonprofit online career counseling test (*berufsprofiling.de*). In this test, participants are asked questions on their personality traits as well as vocational interests. The test takes approximately 35 min to complete. After completing the test, participants received individualized feedback on further academic pathways.

In September 2018, we invited young adults who participated in the former counseling test to take part in a research panel study. We refer to this as our measurement occasion T1. If they agreed, participants were reached out for via E-Mail approximately once a year. Moreover, a financial compensation with proceeding measurement occasions worth € 5,– to € 10,– was provided. Written informed consent was obtained for all participants at all times. Ethical approval for the research project was received and data analyzed in the current study have not been analyzed or published elsewhere before. More information about GePP can be found on OSF: https://osf.io/7w9yj/.

### Sample and attrition effects

For the present study, four measurement occasions are used from the GePP. In September 2018, 1,679 participants of the counseling test agreed to take part in the panel study from which 1,348 provided data on the variables of interest (T1). One year later, at T2, all participants of the counseling test were contacted again. From the 1,089 participants who filled out the test at T2, 804 had also participated at T1 whereas 285 participated for the first time. Data for T3 were collected between April and June 2020 from a total of 902 individuals who had also participated at T1, T2, or both. Data for T4 were obtained in November 2020 from a total of 582 individuals who had participated at T1, T2, T3 or at all of the three measurement occasions.

To check for attrition effects, we examined differences in average life satisfaction by comparing participants who continued versus dropped out on our panel study with proceeding measurement occasions. At T1, average life satisfaction of continuers (participated at T2) did not differ significantly from dropouts (did not participate at T2) (*t*[1271] = -1.61, *p* = 0.11). Also on T2 (*t*[1044] = -0.32, *p* = 0.75) and T3 (*t*[552] = -0.54, *p* = 0.59) continuers (participated at T3 [T4]) and dropouts (did not participated at T3 [T4]) did not significantly differ in their expression of subjective life satisfaction.

We excluded participants who negated the diligence criterion at the end of the survey (Did you work conscientiously on the test?") at the measurement occasions T1 and T4. Participants were informed that their answer had no impact on their financial compensation. We excluded 41 (3.0%) participants from all further analyses on T1 and 3 more participants (0.48%) on T4.

The final sample consisted of *N* = 1,920 participants (T1: *M*age = 19.2, *SD*age = 2.4, range 14 – 28 years) from whom were 65% female. Regarding occupation, at T4, 17% stated to be in a working position, 15% entered a trainee ship, 13% of the participants indicated to do something else or taking a gap year and the majority of 55% were in university or conducting a dual university degree.

#### Measures

##### Life satisfaction

**Fig. 1:**
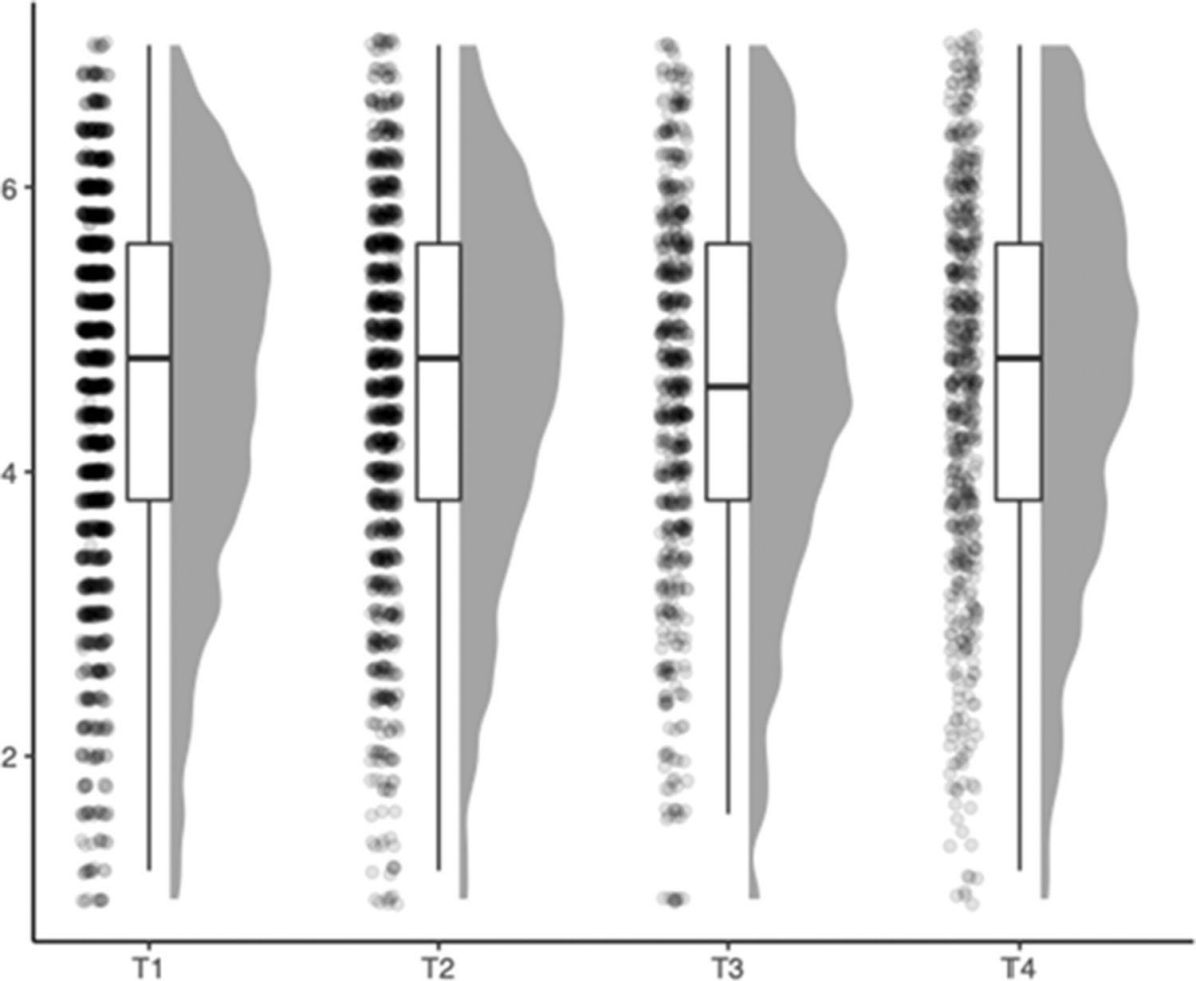
Boxplots of manifest average life satisfaction across four measurement occasions (T1–T4)

Life satisfaction was assessed with the German version of the Satisfaction with Life Scale (SWLS; Glaesmer et al., 2011). The SWLS is the most commonly used measure for life satisfaction and contains 5 items (e.g., “In most ways, my life is close to ideal”). Responses are given on a 7-point rating scale ranging from "does not apply at all" (1) to "partly" (4) to "fully applies" (7). The test was applied at all measurement occasions. The SWLS is found to be highly reliable across all measurement occasions T1 – T4 (α = [0.85 – 0.86, ω = [0.85 – 0.87]). The development and distribution of average manifest life satisfaction can be found in Fig. 1.

##### Self-efficacy

To assess Self-Efficacy, we used the General Self-Efficacy Scale (GSE), a 10-item measure by Schwarzer and Jerusalem (1995). Responses are given on a 7- point rating scale ranging from "does not apply at all" (1) to "partly" (4) to "fully applies" (7). At our measurement occasion T1, the GSE is found to be highly reliable (α = 0.84, ω = 0.84).

##### Subjective perception of the coronavirus pandemic

We assessed the perceived valence of the coronavirus pandemic at two measurement occasions. For T3, data were collected at the beginning of the pandemic between March and June 2020. For T4, we collected data approximately half a year later, in November 2020. Referring to the coronavirus pandemic as a critical life event, we asked participants to rate the two valence items “The critical life event is positive” and “The critical life event is negative”. On T4, the items were slightly changed into “The coronavirus pandemic is positive” and “The coronavirus pandemic is negative”. For each of the items, responses are given on a 7-point rating scale ranging from "does not apply at all" (1) to "partly" (4) to "fully applies" (7). All descriptive statistics and correlations can be found in Table 1.

**Table 1:** Correlations and descriptive statistics among manifest variables

					Correlations							
Variables	*N*	*M*	*SD*	1	2	3	4	5	6	7	8	9
1. LS–T1	1,348	4.57	1.30	*.86*								
2. LS–T2	1,061	4.60	1.28	.64	*.85*							
3. LS–T3	902	4.55	1.33	.62	.73	*.87*						
4. LS–T4	582	4.65	1.35	.62	.71	.78	*.86*					
5. SE	1,486	4.63	.81	.33	.25	.27	.27	*.84*				
6. P–T3p	900	2.64	1.37	.05	–.04	.04	–.04	.03				
7. P–T3n	900	2.57	1.34	.00	–.02	.02	.02	.08	.56			
8. P–T4p	582	2.37	1.46	.03	–.04	–.07	–.02	.03	.37	.31		
9. P–T4n	582	2.45	1.45	.04	–.05	.01	.02	.02	.30	.41	.60	

*Note. N* sample size, *M* mean, *SD* standard deviation, *LST1 – LST4* life satisfaction at measurement occasion T1, T2, T3, and T4, *SE* self-efficacy at measurement occasion T1, *P–p/P–n* perceived negative or positive valence of the corona pandemic at T3 and T4, Coefficient omega is presented in the diagonal

### Analyses

All statistical analyses were performed in R version 4.0.3 (R Core Team, 2019) using the packages psych (Revelle, 2018), lavaan (Rosseel, 2012), semTools (Jorgensen et al., 2022) and ggplot2 (Wickham, 2016).

### Measurement invariance testing

Comparing means and covariances across measurement occasions requires establishing measurement invariance (Vandenberg & Lance, 2000). Thus, to confirm that life satisfaction scores observed at different measurement occasions reflect the same level of the underlying latent variable, we tested for measurement invariance across the four-wave longitudinal data. To do so, progressively more constrained models are compared to each other. We inspected the fit indices comparative fit index (CFI), root mean square error of approximation (RMSEA) and standardized root mean square residual (SRMR). As thresholds, sufficient model fit was assumed when CFI values were 0.90 or greater and both RMSEA and SRMR values were found to be 0.06 or lower (Cheung & Rensvold, 2002; Hu & Bentler, 1999; Marsh et al., 2005).

First, we tested the latent construct of life satisfaction for configural invariance, allowing all factor loadings and item intercepts to vary freely across measurement occasions. This model was then compared to the metric invariance model, where we fixed the factor loadings for each indicator to be equal across measurement occasions. Finally, for strong invariance, the intercepts of the manifest variables were constrained to be equal across measurement occasions. For the present study, strong measurement is a pre-requisite to interpret latent means in life satisfaction across different measurement occasions (Newsom, 2015).

### Statistical analyses

**Fig. 2:**
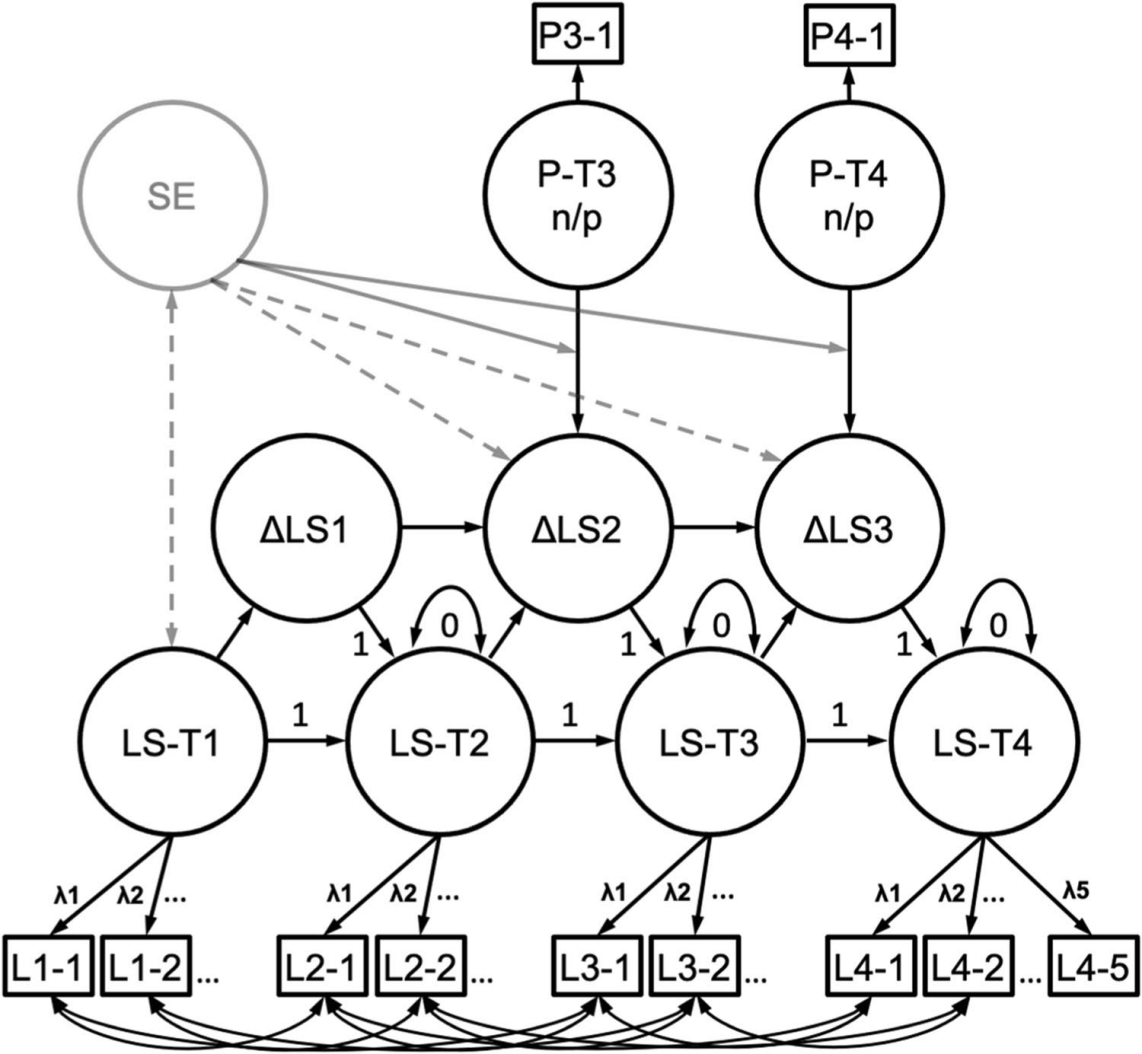
Schematic model of the multiple-indicator latent change score model for the influence of the perception of the corona pandemic. The lower part represents the measurement invariance model for Life Satisfaction (LS) and the upper part for the latent change in Life Satisfaction (ΔLS). Straight arrows show loadings and regression coefficients, curved arrows co-variances. The latent construct of Life Satisfaction was measured at four measurement occasions (T1, T2, T3 and T4), using five manifest indicators each time (L1–L5). The latent variables for the perception of the corona pandemic are indicated by single manifest variables (P3–1 and P4–1). The latent regressions from the perception of the critical life event (P–n/p) to ΔLS reflect the influence of the perception of the corona pandemic on latent change in Life Satisfaction on T3 and T4. SE indicates the influence of self-efficacy on change in latent Life Satisfaction (dotted line) and the moderator effect of self-efficacy on the influence of perception of the critical life event on change in latent Life Satisfaction (grey line)

We used a multiple indicator latent change score model to estimate change in life satisfaction over time. As shown in the schematic model in Fig. 2, the 5 items from the Satisfaction With Life Scale served as manifest indicators for the latent construct and their residuals were allowed to correlate among the sets of repeated measurements. The autoregressive paths between LS–T4 ➔ LS–T3, LS–T3 ➔ LS–T2, and LS–T2 ➔ LS–T1 were fixed to one. To account for change across time, three latent change variables Δ*LS1,* Δ*LS2,* and Δ*LS3* were added to the model. The intercepts and variances of latent life satisfaction (except for LS–T1) were constrained to 0, whereas the latent intercepts and variances of Δ*LS1,* Δ*LS2,* and Δ*LS3* were freely estimated. Moreover, the loadings of Δ*LS3* (Δ*LS2,* Δ*LS1*) on latent life satisfaction at time LS–T4 (LS–T3, LS–T2) were fixed to 1.

Next, to account for the perception of the coronavirus pandemic, we added a latent variable for positive and negative valence at T3 and T4. To do so, we standardized the valence-items prior to inclusion into the model and added them as single indicator latent variables. The covariates’ error variance was set to 0.20 which equals an estimated reliability of 0.80 (Schumacker & Lomax, 2004). Previous studies showed that perceived valence constitutes a complex dimension, and that positive and negative affect should be interpreted distinct from each other (Dejonckheere et al., 2021; Luhmann et al., 2020; Zammitti et al., 2021). Thus, we analyzed the models separately for self-reported positive and negative perception of the coronavirus pandemic. The change score *ΔLS2* was then regressed on the latent covariate *perception of the life event P–T3.* Analogously, the same was done for *ΔLS3* ➔ *P*–*T4.*

Next, we added the variable self-efficacy to the model. We used data from trait self-efficacy at the first measurement occasion. At T1, the COVID-19 disease did not yet exist and could therefore not interfere with the self-efficacy report. To assess the influence of self-efficacy on the development of life satisfaction with respect to the coronavirus pandemic, we regressed the latent change scores *ΔLS3* and *ΔLS4* on self-efficacy*.* Moreover, life satisfaction at T1 and self-efficacy were allowed to correlate (as shown in Fig. 2 by the arrow with two heads), indicating a baseline relation between life satisfaction and self-efficacy.

Finally, we investigated whether self-efficacy moderated the influence of perception of the life event on changes in life satisfaction. As product indicator methods provide an accurate method to estimate and test latent interactions (Schoemann & Jorgensen, 2021), the moderators were built with an interaction term between the standardized variable *perception of the life event* and the self-efficacy measure. The life satisfaction change factors *ΔLS3* and *ΔLS4* were then regressed on the moderator, respectively.

We accounted for missing values data by using Full Information Maximum Likelihood (FIML). Values were missing when, for examples, participants did not respond on all measurement occasions or did not finish the entire questionnaire. Under multivariate normality, FIML maximizes the utility of all existing data, decreases bias and increases statistical power compared to omitting incomplete cases (‘complete case analysis’; Baraldi & Enders, 2010; Kievit et al., 2018).

## Results

The configural invariant model of life satisfaction showed good fit (χ^2^ = 234, *df* = 134, CFI = 0.988, TLI = 0.983, RMSEA = 0.023, SRMR = 0.038) and the weak invariant model revealed similar fit (χ^2^ = 257, *df* = 146, CFI = 0.987, TLI = 0.983, RMSEA = 0.023, SRMR = 0.043). Likewise for the strong invariant model, the data fitted the model well (χ^2^ = 299, *df* = 161, CFI = 0.985, TLI = 0.982, RMSEA = 0.024, SRMR = 0.043). To account for the sensitivity of the χ^2^ difference test to sample size exceeding 300, we relied on fit parameter criteria proposed by Cheung and Rensvold (2002): a comparative fit index (CFI) difference not larger than 0.01 across models implies that the model fit does not deteriorate considerably. Therefore, strong measurement equivalence was accepted for the life satisfaction measure (ΔCFI < 0.010) which allows for analyzing mean differences across measurement occasions. Therefore, all further analyses are based on this model. The results of the measurement invariance testing are depicted in Table 2.

**Table 2:** Fit indices for measurement models with increasing degrees of invariance across time

Model	χ2 (*df*)	*p*(χ2)	CFI	TLI	RMSEA	RMSEA 90% CI	SRMR
Model 1: Configural invariance	234 (134)	< .001	.988	.983	.023	[.018 – .028]	.038
Model 2: Metric invariance	257 (146)	< .001	.987	.983	.023	[.019 – .028]	.043
Model 3: Strong invariance	299 (158)	< .001	.985	.982	.024	[.020 – .028]	.043

*Note*. *Χ*^*2*^ chi-square difference statistic, *Df* degrees of freedom, *CFI* comparative fit index, *GFI* goodness of fit index, *RMSEA* root mean square error of approximation, *RMSEA 90% CI* 90% confidence interval of RMSEA, *SRMR* standardized root mean square residual

### Hypotheses testing

**Fig. 3:**
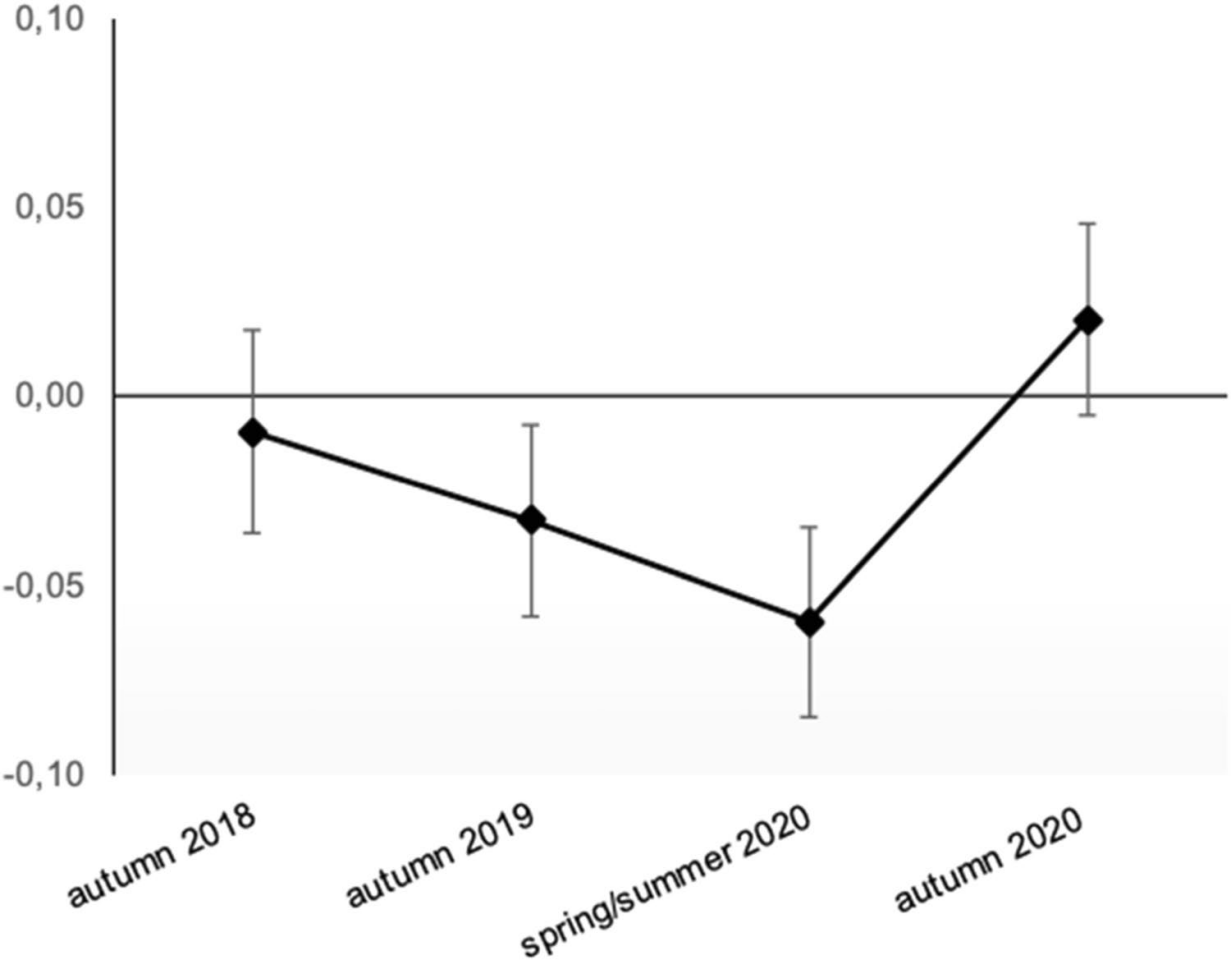
Standardized latent life satisfaction across four measurement occasions (T1–T4)

For the development of life satisfaction, the multiple indicator latent change score model showed good model fit (χ^2^ = 495, *df* = 158, CFI = 0.984, RMSEA = 0.025, SRMR = 0.044). Although there was a tendency in the expected direction of latent life satisfaction (Fig. 3) towards a decrease on T3–the onset of the preventive COVID-19 measures–the effect was not significant for neither of the latent (standardized) intercepts (*ΔLS1: est* = -0.02*, se* = *0.04, p* = 0.51; *ΔLS2 est* = -0.03*, se* = *0.0*4*, p* = 0.52; *ΔLS3*, *est* = 0.08*, se* = 0.05*, p* = 0.07*)*. Thus, and contrary to previous findings, we rejected our first hypothesis that restrictions lead to a general change in self-reported average life satisfaction.

**Fig. 4:**
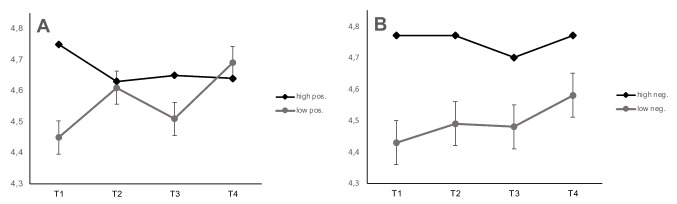
**a**. Average life satisfaction splitted by high versus low positive perception of the corona pandemic across four measurement occasions (T1–T4). **b**. Average life satisfaction splitted by high versus low negative perception of the corona pandemic across four measurement occasions (T1–T4)

With respect to the second research question, our findings revealed a more nuanced picture. To test whether perceptions of the COVID-19 related restrictions predict change in life satisfaction, we separately added latent variables reflecting perceived negative and positive valence. Again, the model fitted the data well (χ^2^ = 336, *df* = 194, CFI = 0.984, RMSEA = 0.023, SRMR = 0.043). When asking participants how positively they experienced the coronavirus pandemic, they showed significant change in life satisfaction with the onset of the anti-COVID-19 measures at T3 (*est* = 0.09, *se* = 0.04, *p* = 0.03). Specifically, people who experienced the coronavirus pandemic less positively, showed a stronger decline in their levels of life satisfaction with the onset of the preventive measures at T3 (Fig. 4a). At T4, when the anti-COVID-19 measures were relaxed again, there was no significant effect of a positive perception (*est* = 0.07, *se* = 0.03, *p* = 0.06). Contrary, when asking participants how negatively they experienced the coronavirus pandemic, no significant influence of the perception of the life event on change in latent life satisfaction was noted (Fig. 4b), (T3: *est* = 0.07, *se* = 0.05, *p* = 0.25; T4: *est* = 0.02, *se* = 0.03, *p* = 0.95).

**Fig. 5:**
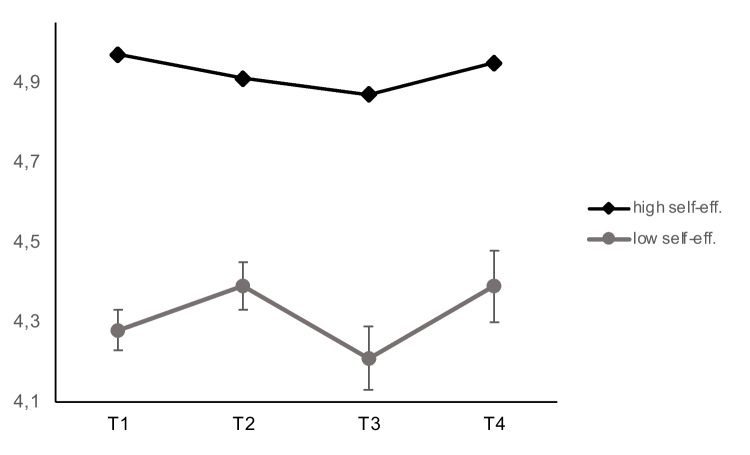
Average life satisfaction with regard to high versus low levels of self-efficacy across four measurement occasions (T1–T4)

Further, we tested whether self-efficacy as a protective factor might lessen the impact of COVID-19 related restrictions on life satisfaction. We conducted our analyses in consecutive steps. First, we tested for a main effect of self-efficacy and added self-efficacy to the model. The data fitted the model well (χ^2^ = 355, *df* = 210, CFI = 0.984, RMSEA = 0.021, SRMR = 0.041). Self-efficacy showed a significant effect on latent change in life satisfaction at T3 (*est* = 0.13, *se* = 0.05, *p* = 0.02). At this measurement occasion, people high in self-efficacy showed almost no change in life satisfaction, whereas life satisfaction dropped for young adults with low self-efficacy (Fig. 5). Moreover, we found a significant latent covariance of self-efficacy and latent life satisfaction at T1 (*est* = 0.44, *se* = 0.04, *p* < 0.001). As depicted in Fig. 5, participants with high levels of self-efficacy displayed also higher levels of life satisfaction from the beginning of our study.

Second, we tested for a moderating effect of self-efficacy. Adding a moderating variable to the model showed similar fit (χ^2^ = 401, *df* = 246, CFI = 0.983, RMSEA = 0.020, SRMR = 0.041). However, as depicted in Table 3, neither for perceived positive (T3: *est* = 0.02, *se* = 0.04, *p* = 0.61; T4: *est* = -0.05, *se* = 0.05, *p* = 0.35), nor for perceived negative valence (T3: *est* = -0.03, *se* = 0.04, *p* = 0.47; T4: *est* = -0.02, *se* = 0.05, *p* = 0.61), the moderator showed a significant effect on latent change in latent life satisfaction. Thus, the influence of the perception of the corona-crises on changes in life satisfaction was not moderated by expressions of self-efficacy.

**Table 3:** Model fit parameters and estimates for the latent univariate change score model (see Fig. 1), N = 1,944

Model		χ2(df)	*p*(χ2)	CFI	TLI	RMSEA	RMSEA 90% CI	SRMR	ΔLS1	ΔLS2	ΔLS3	
H1		295 (158)	< .001	.984	.981	.025	[.020 – .029]	.044	–.024	–.025	.081	
									P-T3p➔ΔLS2	P-T3n➔ΔLS3	P-T4p➔ΔLS2	P-T4n➔ΔLS3
H2		336 (194)	< .001	.984	.981	.023	[.019 – .027]	.043	.091*	.071	.066	.018
									SE➔ΔLS2	SE➔ΔLS3		
H3		299 (161)	< .001	.985	.981	.024	[.020 – .028]	.043	.130*	.095		
									ModT3p➔ΔLS2	ModT3n➔ΔLS3	ModT4p➔ΔLS2	ModT4n➔ΔLS3
									.021	–.046	–.031	–.024

*Note. Χ*^*2*^ chi-Square value, *Df* degrees of freedom, *CFI* comparative fit index, should be above .90, *RMSEA* root mean square error of approximation, should be below .08, *SRMR* standardized root mean square residual, should be below .05, *Δ-LS* change in life satisfaction, *V-p/n ➔ ΔLS* latent regression coefficient of perceived positive (p) or negative (n) valence of the corona pandemic on latent change in life satisfaction, *SE ➔ ΔLS* latent regression coefficient of self-efficacy on latent change in life satisfaction, *MODp/n ➔ ΔLS* latent regression coefficient of the moderator for positive (p) and negative (n) valence on latent change in life satisfaction at T3 and T4

^*^p < .05. ** *p* < .01. ***p < .001

## Discussion

The preventive measures to reduce the spread of the COVID-19 disease have drastically altered young adults’ lives. The current study focused on the dynamics of subjective life satisfaction and the collectively experienced critical life event, the coronavirus pandemic. We provide comprehensive information on data before and during the pandemic, including separate measurement occasions during a strict lockdown and when the implemented restrictions were relaxed again. Although no general decline in life satisfaction was noted, we found profound differences in interindividual change when accounting for the perception of the pandemic. Further, we found a main effect of self-efficacy on life satisfaction at T3, the onset of the anti-COVID-19 measures. However, expressions of self-efficacy were unrelated to the influence of the perception of the pandemic on self-reported life satisfaction.

### Mean change in life satisfaction

In the current sample of young adults in Germany, no general change in subjective life satisfaction with regard to the measures to prevent the spread of COVID-19 was found. Despite a tendency towards a decline at the onset of the coronavirus pandemic and a tendency towards an increase half a year later, participants’ levels of average life satisfaction were not significantly different across the four measurement occasions. There might be several possible explanations. First of all, past research has shown that life satisfaction constitutes a rather stable construct which is not easily affected by environmental influences (e.g., Headey & Wearing, 1989; Myers & Diener, 2018). Mean levels of life satisfaction tend to vary little across the life span and only heavy disruptions provoke lasting change in subjective life satisfaction (Clark et al., 2008; Lucas et al., 2004). This stands in line with the assumptions of the earlier described Set Point Theory. Set Point Theory proposes that people have a determined set point for their personality traits and that only decisive environmental influences can permanently change this set point (Anusic et al., 2014; Lucas et al., 2004; Yap et al., 2012). Further, the underlying key mechanism of Set Point Theory is adaptation. Thus, on the one hand, the implementations to reduce the spread of the coronavirus pandemic might not have been decisive enough, to evoke far reaching changes in young adults’ overall attitude towards one's life. On the other hand, young adults might have successfully and quickly adapted to the stressors of the pandemic. Thus, they might have figured out a way of coping without having their average life satisfaction being influenced lastingly, or at least not as long lasting so that the measurements used in the current study could have detected such change.

Furthermore, in a large German panel data set, van Praag and colleagues (Praag et al., 2003) identified the most important determinants of overall life satisfaction: health, family, and finances. In terms of health, there were fewer identified cases of COVID-19 in children and young adults, they experienced less severe courses of infection and fewer deaths were reported (WHO, 2021). Regarding family, young adults still depend on their social home environments, thus, in comparison to many other individuals, a reduction of social contacts might not have directly resulted in isolation. Moreover, most of young adults still rely on financially dependencies to e.g., parents. Therefore, they might have experienced possible financial repercussions merely as indirect stressors.

Another explanation might be related to inter-individual differences in the volatility of life satisfaction and the mere assessment of average life satisfaction. Headey and Muffels (2018) investigated dynamics in life satisfaction over 25 years in Germany and found that some people experience a lot of volatility in subjective life satisfaction, even though their overall mean level of life satisfaction changes barely over time. For example, high levels of neuroticism are associated with low average life satisfaction but high volatility. Further, along with other aspects such as behavioral choices and socio-economic characteristics, life satisfaction volatility seems to be also related to age, with its highest variability in teenage years (Headey & Muffels, 2018). With regard to the coronavirus pandemic, forced restrictions might have influenced young adults on very different dimensions in daily living. Since the beginning of the pandemic, a great number of studies reported declines in closely related constructs to well-being. For example, in a large longitudinal study, Lee et al. (2020) reported increases in loneliness in young adults with the onset of the pandemic. Likewise, using data from a large-scale daily diary study in Germany, Buecker and Horstmann (2021) found that the quality of social relationships was perceived worse during compared to before the pandemic. Moreover, the numbers of studies reporting psychological distress, mental health problems as well as socio-economic implications for young people seem endless (e.g., Nicola et al., 2020; Ren et al., 2021, Rogowska et al., 2020; Wang et al., 2020; Xiong et al., 2020; Zacher & Rudolph, 2021). Therefore, overall life satisfaction levels may not reflect experiences during the pandemic precisely enough and even if the subordinate expressions of average life satisfaction remain stable, young adults still might have experienced phases of distress. Additionally, the country of the current sample should be taken into consideration. Anti-COVID-19 measures may be less strict than in other countries, where, for example, much stricter mobility restrictions like curfews were imposed (e.g., in Italy and China). Together, these examples illustrate how important it is to look out for underlying processes which might account for change in well-being and individual life satisfaction.

### Subjective perception of the coronavirus pandemic

Correspondingly, our results revealed a different picture when we asked participants how they perceived the coronavirus pandemic. With regard to our second research question, we found a significant effect for positive valence on life satisfaction. According to how positively participants perceived the pandemic, they experienced a differential change in subjective life satisfaction at the onset of the restrictions. More specifically, people who stated they experienced the coronavirus pandemic less positively, showed a stronger decline in their levels of life satisfaction at the beginning of the anti-COVID-19 measures. Interestingly, this group of participants displayed a greater increase of life satisfaction on T4, even slightly above those, who experienced the coronavirus pandemic on average more positively (see Fig. 4a). Presumably, it seems like the relief was stronger for those who struggled more, resulting in a slight boost of subjective life satisfaction. Compared to the harsh lockdown spring 2020, in autumn 2020 at the time of T4, a lot of restrictions were lifted in Germany, with open schools and restaurants, and social contact, allowing young adults’ lives to normalize.

Contrary, we found no significant effect regarding negative valence. Thus, the degree of negative perception did not predict changes in life satisfaction. Nonetheless these results should be interpreted with caution. The scope of the present study only enables to highlight short-term consequences of the coronavirus pandemic, whereas negative long-term effects on well-being and especially mental health are still unknown. Moreover, age and current life stages have implications for how an individual can perceive events (Cohen et al., 2019). Thus, future research should compare the results of the current study to different age groups and account for external additional stressors beyond the coronavirus pandemic.

As such, our results illustrate once more the importance to differentiate between the assessment of the mere occurrence of a critical life event and how people perceive them. Our findings stand in line with previous research which has demonstrated that trajectories corresponding personality trait change (de Vries et al., 2021; Haehner et al., 2021; Rakhshani et al., 2021), subjective well-being, and mental health (Fassbender et al., 2021; Luhmann et al., 2020) show different patterns when accounting for a person’s perception of an event. In the current study, our findings on perceived valence imply a direct association of how people perceive the restrictions of the pandemic and their subjective well-being. Apparently, young adults show individual differences in dealing with and adapting to the pandemic which gives ground for our third research question– addressing internal resources people might rely on when facing critical life events.

### The impact of self-efficacy

Past research demonstrates the need to consider potential moderators of the relationship between event perceptions and personality traits, and to explore how certain associations differ between people (e.g., Haehner et al., 2021; Rakhshani et al., 2021). Since internal resources have been shown to influence coping with the pandemic (Rogowska et al., 2020), we focused on a personality trait that might protect from negative effects of the anti-COVID-19 measures. Thus, we investigated whether self-efficacy acts as a protective factor and buffers the negative influence on life satisfaction. Interestingly, self-efficacy showed a direct effect on delta life satisfaction at T3 when the preventive COVID-19 measures were first implied. Moreover, our results indicated that young adults with high versus low expressions of self-efficacy differ in their overall level of life satisfaction from the beginning of our study. However, self-efficacy did not moderate the relationship between the perception of the coronavirus pandemic (either positive or negative) and related changes in life satisfaction.

Since self-efficacy describes the inherent belief to be able to rely on one’s competencies, people might feel more capable to overcome the obstacles related to and associated with the anti-COVID-19 measures. It has been shown that people with a strong sense of self-efficacy also have the ability to regulate cognitive, motivational, and affective processes more easily (Bandura, 1977). With regard to changes in life satisfaction, research suggests that people typically experience periods in life where they are increasingly happy or increasingly unhappy (Headey & Muffels, 2018). Correspondingly, it can be argued, that self-reported life satisfaction stays rather stable, even in the face of critical life events because people high in self-efficacy are able to process the implementations of the pandemic more effectively. For example, they might “rise up” to the concrete challenge when working from home and mastering children day care at the same time. With regard to young adults, this could imply keeping up with social contacts by enforcing online meetups, maintaining physical exercises and hobbies, or adapting to the anti-COVID-19 measures in creative ways. In turn, this task could be responsible for periods in which their life satisfaction moves above or below the individual long term mean of life satisfaction but without triggering a prolonged disruption.

In sum, our results highlight the importance of reporting trajectories in life satisfaction with regard to the preventive COVID-19 measures, the perception of such events as well as internal factors that influence these. While we found a main effect of the perception of the pandemic and self-efficacy, no average decline in life satisfaction was noted. However, one should not underestimate the impact of the coronavirus pandemic, as research across various domains have shown how the pandemic interferes with individual psychological well-being (e.g., Armour et al., 2020) and long-term consequences on general well-being are still unknown. To systematically develop effective intervention strategies, research should focus on underlying mechanisms of how and why people cope differently with the implementations of the coronavirus pandemic, and to identify vulnerable groups of individuals. With ongoing high numbers of infections and related restrictions on daily living, our results help identifying possible buffering effects to maintain psychological well-being of young adults.

## Limitations and future directions

The current results shed light on the distinctive nature of the perception of critical life events. However, despite positive and negative valence, more characteristics of life events should be addressed in future research. To account for a whole picture on the influence of critical life events, for example, the predictability, impact, emotional significance, and challenge could be assessed (Kendler et al., 2003; Luhmann et al., 2020). With proceeding measurement occasions, we considered the most recent research on the assessment of life events but at our measurement occasion T3, no other information than perceived valence was assessed. Therefore, for reasons of comparability to T4, we focused on perceived positivity and negativity of the coronavirus pandemic.

Moreover, there are some limitations concerning our sample. Even though the sample was quite large, we only examined trajectories of life satisfaction for young adults in Germany that self-selected in our panel study. This sample might differ from other populations in the perception of the coronavirus pandemic, since other countries were exposed to a harsher lockdown and even more restrictions on daily living. As the coronavirus pandemic effected young adults all over the world, it would be fruitful for future research in this area to review and compare our findings on subjective well-being to other communities and cultures except for western industrialized countries such as Germany. Moreover, caution should be taken when comparing our results to other findings, since the effects of the perception of the coronavirus pandemic and self-efficacy on life satisfaction were yet significant but rather small. Further, our sample consisted predominantly of female participants. Although we gathered data from diverse participants all over Germany, the higher percentage of female young adults might be explained by self-selection to voluntary participate in a research study.

Further, we assessed self-efficacy at the beginning of our investigation. The benefit of this approach is that the self-efficacy measure was unrelated to possible interfering coronavirus content. However, self-efficacy constitutes a trait which is developing with time. Thus, levels of self-efficacy might have changed during our investigation over a period of three years and recent self-efficacy might have changed accordingly.

## Conclusion

The present study is one of the first analyzing comprehensive longitudinal data on life satisfaction with assessments before and during the pandemic, as well as when tight anti-COVID-19 measures were relaxed again. We aimed at investigating how the coronavirus pandemic influences trajectories in life satisfaction in young adults. Life events such as the coronavirus pandemic are complex and perceived quite differently. Therefore, we disentangled associations between the perception of the coronavirus pandemic and the personality trait self-efficacy which might buffer the influence of the restrictions on subjective life satisfaction. While we found no evidence for a general decline, perceived positive valence and self-efficacy were associated with change in life satisfaction at the onset of the preventive COVID-19 measures. Our results imply that life events encompass meaningful changes in individual well-being and more studies are needed to enrich practical implications to deal with the consequences of the pandemic.

## Data Availability

All data are available at OSF repository, https://osf.io/7w9yj/
